# Carbohydrate-restricted Diet and Exercise Increase Brain-derived Neurotrophic Factor and Cognitive Function: A Randomized Crossover Trial

**DOI:** 10.7759/cureus.5604

**Published:** 2019-09-09

**Authors:** Amy Gyorkos, Mark H Baker, Lauren N Miutz, Deborah A Lown, Michael A Jones, Lori D Houghton-Rahrig

**Affiliations:** 1 Preventive Medicine, Biological Sciences, Western Michigan University, Kalamazoo, USA; 2 Exercise Science, Grand Valley State University, Allendale, USA; 3 Preventive Medicine, Kinesiology, University of Calgary, Calgary, CAN; 4 Preventive Medicine, Biomedical Sciences, Grand Valley State University, Allendale, USA; 5 Physical Medicine and Rehabilitation, Western Michigan University, Kalamazoo, USA; 6 Preventive Medicine, College of Nursing, Grand Valley State University, Allendale, USA

**Keywords:** bdnf, neurotrophic factor, metabotrophin, low carbohydrate diet, high intensity interval training, metabolic syndrome, paleolithic diet, cognitive functioning, restrictive carbohydrate diet, ketogenic

## Abstract

Introduction

Metabolic syndrome (MetS) has been recognized as one of the most important clinical challenges and global health issues of today. Growing evidence suggests that mechanisms of energy metabolism may also play a key role in mediating aspects of cognitive function. Brain-derived neurotrophic factor (BDNF) is one such factor well known for its critical role in neuronal plasticity, including memory and learning, and more recently metabolic processes. BDNF levels have been shown separately to be dependent on diet and exercise programming.

Purpose

The purpose of this study was to investigate the effect of diet and exercise on BDNF levels and cognitive functioning with any metabolic association in individuals characterized with MetS.

Methods

Twelve subjects with MetS followed a randomized crossover design with two four-week interventions, including a carbohydrate (CHO)-restricted Paleolithic-based diet (CRPD; <50gCHO) with sedentary activity (CRPD-Sed) and CRPD with high intensity interval training (HIIT; CRPD-Sed), separated by a four-week washout period. The HIIT exercise consisted of 10 x 60 s cycling intervals interspersed with 60 s of active recovery 3 day/week for four-week. Serum BDNF was detected and quantified via enzyme-linked immunosorbent assay (ELISA). Cognitive executive function (Stroop Test) and self-perceived cognitive symptoms and function (MOS-CFS) were quantified. A two-way analysis of variance with repeated measures was performed with post-hoc analysis using simple effects analysis with a Bonferroni adjustment. The level of statistical significance was established a priori as P < 0.05.

Results

Compared to baseline, CRPD-Sed and CRPD-Ex improved variables for cognitive function, including increased peripheral serum BDNF levels (20% and 38%), psychomotor speed and cognitive flexibility (-14%, -14%), and self-perceived cognitive symptoms and functioning (+8%, +16%), respectively. BDNF inversely correlated with %body fat (r = -0.35, P < 0.05), fasting glucose (r = -0.64, P < 0.05), triglycerides (r = -0.55, P < 0.05), and insulin sensitivity (r = -0.25, P < 0.05).

Conclusion

This study shows the short-term beneficial effects of carbohydrate-restricted diet on serum BDNF and executive function in those individuals characterized with MetS. We have shown that the addition of exercise can further improve neuroprotection and cognitive function beyond the results of diet alone.

## Introduction

Brain-derived neurotrophic factor (BDNF) is a member of the neurotrophin family consisting of small secreted proteins that play a critical role in the development, maintenance, survival and plasticity of the central and peripheral nervous system [1]. More recently, several lines of evidence indicate that BDNF and its high affinity receptor, tropomyosin-related kinase B (TrkB), are critical players in metabolic processes, including body weight control, food intake and energy homeostasis. BDNF and TrkB receptor expression have been identified in the two major integrative autonomic centers involved in energy homeostasis; the hypothalamus and the dorsal vagal complex as well as peripheral tissues, such as skeletal muscle, heart, liver and adipose tissue [2, 3]. Therefore, the term “metabotrophins” (Greek for “nutritious for metabolism”) has emerged signifying the close connection between BDNF and the metabolic system [4].

Hyperphagia, obesity, reduced satiety and metabolic imbalances have been observed in BDNF deletions, BDNF knockout in rodents, and BDNF haploinsufficiency in humans [5-7]. Further, researchers have shown that circulating BDNF is lower in individuals with metabolic syndrome, obesity and type II diabetes mellitus (T2DM) when compared to healthy controls [8, 9]. Conversely, BDNF infusions were able to attenuate or reverse weight gain, hyperglycemia, hyperphagia and obesity [10].

Lifestyle factors such as diet and exercise have also shown an effect on circulating BDNF levels. The typical western diet, consisting of high-fat and high-carbohydrate (CHO) intake, reduces hippocampal BDNF content, while nutritional restriction increases brain BDNF content [11, 12]. In addition, acute bouts of exercise have consistently increased BDNF in platelets and serum in healthy individuals [13]. BDNF levels seem to be intensity dependent with high intensity training elevating serum BDNF more than moderate intensity [14].

It is therefore the purpose of this study to investigate the effects of a carbohydrate-restricted Paleolithic-based diet (CRPD) with and without high intensity interval training (HIIT) exercise on circulating BDNF levels, functional cognition, cardio-metabolic factors and the relationship between variables.

## Materials and methods

Subjects and study design

**Figure 1:**
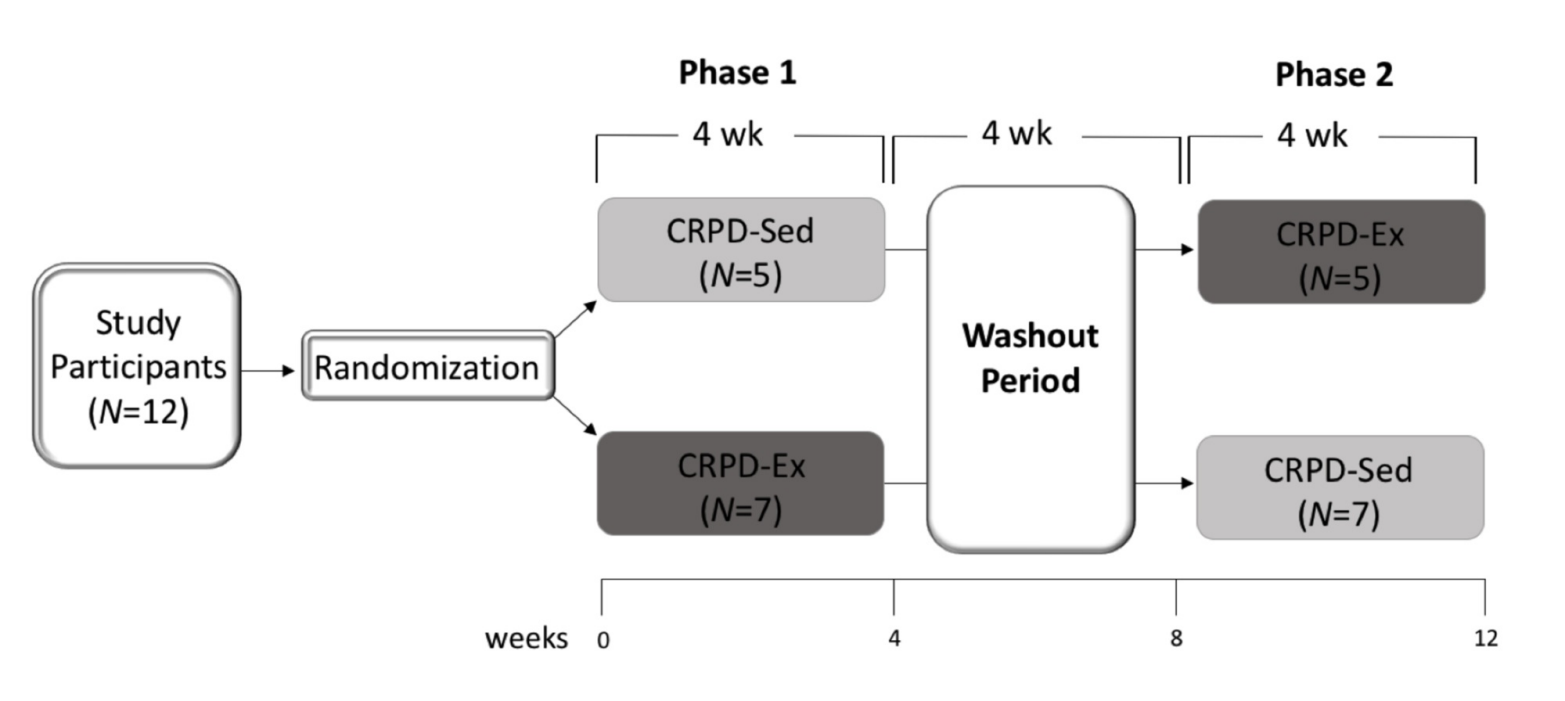
The experimental design is a randomized, two-phase crossover trial with a washout period. Subjects characterized with MetS (N = 12) were randomly assigned to one of two arms of the study (CRPD-Sed or CRPD-Ex). After four weeks, all subjects had a four-week washout period where they returned to baseline diet and exercise behaviors. Following the washout, subjects entered the opposite arm of the study for four weeks. Data collection took place at weeks 0, 4, 8 and 12, which correlated with before and after each phase of the study. N: Number of subjects randomized to that respective group; CRPD-Sed: Carbohydrate-restricted Paleolithic-based diet without exercise; CRPD-Ex: Carbohydrate-restricted Paleolithic-based diet with exercise.

Twelve free-living individuals completed a randomized, two-phase crossover dietary and exercise trial. Subjects followed a crossover design in which each participant received both four-week interventions in randomized order, separated by a four-week washout period to reduce carryover effect (Figure 1). The two phases of the crossover design included 1) carbohydrate-restricted Paleolithic-based diet with sedentary behavior (CRPD-Sed) and 2) carbohydrate-restricted Paleolithic-based diet with high intensity interval training (CRPD-Ex).

This study included men (n = 4) and woman (n = 8) participants (40.9 ± 20.2 years of age) that characterized with metabolic syndrome (MetS) according to the National Cholesterol Education Program-Adult Treatment Panel III [15]. In addition to MetS, inclusion criteria were self-reported to ensure that weight was stable for at least three months and subject could be classified as relatively sedentary (defined as not engaged in physical activity at least 3 days/week for three months). Exclusion criteria were following any special diets, medications for chronic disease, and cardiovascular, metabolic, pulmonary, and osteoarticular disease. The study was conducted in accordance with the guidelines of the Institutional Review Board at Grand Valley State University and all participants provided written consent.

Diet intervention

All subjects were asked to follow a carbohydrate-restricted Paleolithic-based diet (CRPD) that consisted of the following: 1) unlimited amounts of unprocessed lean meat, fish, eggs, leafy and cruciferous vegetables, root vegetables, fruit, and nuts, 2) moderate amounts of nuts, dried fruit, potatoes (<1 medium-sized/d), and wine (<1 glass/d), and 3) devoid of cereal grains, dairy, beans, legumes, refined fats, bakery items, soft drinks, beer, extra salt and sugar. The goal for macronutrient intake was 25, 60, 15% for protein, fat, and carbohydrate, respectively, with CHO remaining under 50 g/d. No calorie parameters were specified.

In order to track macronutrient consumption, subjects were asked to use a journal and a phone app to record food items and quantities. In addition, a five-step multiple-pass 24-hour recall method was used to obtain diet intake following the USDA protocol [16]. The 24-hour recalls indicated that all subjects achieved the diet goals.

Exercise intervention

All subjects were asked to participate in a supervised exercise session three days a week for four weeks during the CRPD-Ex trial. Each session consisted of high intensity interval training (HIIT) on a cycle ergometer, including a 3-min warm-up, 10 x 60s cycling intervals interspersed with 60s of active recovery, and a 3-min cool down. Heart rate was monitored continuously and recorded every 15 minutes to ensure that a full effort was reached indicated by attaining ~90% maximal heart rate (HRmax). The HRmax was determined by a maximal graded exercise test (VO2peak).

VO2peak aerobic testing

The peak oxygen consumption (VO2peak) and HRmax were assessed by a maximum continuous graded exercise test (GXT) on a cycle ergometer. Subjects pedaled at a cadence of 60-80 revolutions per minute at a workload that started at 60 watts and increased every minute by 20 watts until the subject was fatigued. Volitional fatigue was determined by the inability to maintain the required power output despite verbal encouragement and was reached within 8-15 min in all subjects. Researchers confirmed two out of four criteria to ensure maximal effort was given during the test including (a) HR within 10% age-predicted HRmax, (b) plateau in VO2, (c) respiratory exchange ratio of greater than 1.15 and/or (d) RPE of ≥18. Data was averaged over 15-second intervals with the highest VO2 and HR recorded as VO2peak and HRmax, respectively. Water was given ad libitum. All subjects were fully compliant with exercise session participation and HIIT exercise was well tolerated.

Basal serum BDNF

Blood collection was performed at weeks 0, 4, 8 and 12 of the study during the same time of day and at least 72 hours post-exercise. Collected blood was centrifuged (1,500 x g for 15 min) and the serum was aliquoted and frozen at -80°C until analysis. A commercial enzyme-linked immunosorbent assay (ELISA) was used for the detection and quantification of circulating serum BDNF (#DBD00, Thermo Fisher Scientific) content. The 96-well plates were read at 450 nm with an absorbance microplate reader (BioTek Instruments Inc., VT, USA). The sensitivity and intra-assay CV for BDNF was 0.76 pg/ml and 6.1%, respectively.

Serum BDNF was measured in the periphery, which is presumed to originate in the brain, cross the blood-brain barrier and therefore can be measured in the peripheral plasma and serum. BDNF content levels in the periphery and in the brain are similar, including parallel measurements in hippocampal BDNF protein and mRNA [17].

Cognitive speed & flexibility

The Stroop test measures selective attention and cognitive speed and flexibility (executive functions) based on the Stroop effect of interferences in the recognition reaction time to a task. The Stroop test has an “Off” state in which subjects view a neutral stimulus (###) presented in red, green or blue. The Stroop also has an “On” state in which the subjects were presented a stimulus in the form of a word that spelled out a color. The stimulus was randomly delivered as either congruent (e.g., the word “red” was displayed in a red color) or incongruent (e.g., the word “blue” was displayed in a green color). All stimuli were displayed on the screen one at a time. In both the “Off” and “On” states, the goal was to select the color of the stimulus in as little time as possible by touching the matching color word choice at the bottom of the screen. The color choices at the bottom of the screen were randomized and not fixed to their respective positions.

All subjects were given verbal test instructions and two training runs prior to the beginning of each state. All subjects continued until they completed five successful consecutive runs (10 stimuli/run) without any mistakes for the “Off” and “On” state. The total time it took for participants to complete five correct runs in the “On” state and five correct runs in the “Off” state was recorded. The subject’s cognitive speed and flexibility was determined by adding “On” time to “Off” time and cognitive flexibility was determined by subtracting “Off” time from the “On” time.

Cognitive symptoms & function

The Medical Outcomes Study Cognitive Functional Scale (MOS-CFS) was used as a self-report measure of cognitive symptoms and function. Subjects were asked to fill out this six-question, Likert-type scale, assessing memory, reasoning and thinking at weeks 0, 4, 8, and 12 of the study. The responses to individual questions were summed and the score was then converted to a 0-100 point scale (least-most favorable functioning). The scale has been shown to be reliable and demonstrate discriminant validity and responsiveness to clinically meaningful change [18].

Statistical analysis & correlations

All statistical analyses were performed using SPSS 24 (IBM Corp., Armonk, NY) statistical software. Descriptive statistics were calculated to define means and standard errors for all variables. Data were analyzed using a two-way analysis of variance (ANOVA) with repeated measures to evaluate change over time (0, 4, 8, 12 weeks) and condition (CRPD-Sed and CRPD-Ex). Tukey’s post hoc comparison with a Bonferroni adjustment was used to test for statistically significant differences between groups.

Linear regression analysis was performed on the individual samples to evaluate the association between variables. The circulating serum BDNF content was correlated to data previously described for correlative analysis (paper submitted: Gyorkos, 2019). The previously published data involved 1) Body Composition including body mass, waist adiposity, and percent body fat (%BF) and 2) Cardio-Metabolic markers including fasting insulin, fasting glucose, insulin sensitivity (HOMA-IR) and lipoproteins. The lipoprotein profile included total cholesterol, triglycerides (TG), high density lipoprotein cholesterol (HDL-C), and low-density lipoprotein cholesterol (LDL-C).

The level of statistical significance was established a priori as P < 0.05. Values are reported as means ± standard deviation (SD).

## Results

Dietary intake

Subjects showed good compliance with dietary recommendations according to analysis of average dietary intake (Table 1). Dietary macronutrient compositions (carbohydrate, protein and fat) were significantly altered from baselines and indicated a high degree of subject compliance. Evidence of compliance with restricting carbohydrates was indicated by measuring and detecting low-grade blood ketone β-hydroxybutyrate levels (0.53 ± 0.29 mmol/L). Further, no significant changes were observed between baselines (at time 0 and following the washout period). This indicates good compliance returning to pre-study diet composition throughout the washout period. A spontaneous reduction in total caloric intake was observed. Total caloric intake assessed in CRPD-Sed (1306 kcal: %CHO:fat:protein = 16:62:22) differed significantly from baseline (2189 kcal: %CHO:fat:protein = 49:37:15). CRPD-Ex caloric intake (1590 kcal: %CHO:fat:protein = 13:67:22) also differed significantly from baseline (2466 kcal: %CHO:fat:protein = 45:43:13). This spontaneous reduction in caloric intake is similar to other similar carbohydrate-restricted studies in which caloric intake was not regulated [19].

**Table 1:** Average nutrient intake for subjects during baseline and diet interventions. Values are mean ± SD *Significance between baseline and week 4 of intervention at P ≤ 0.05 CRPD: Carbohydrate-Restricted Paleolithic-Based Diet

	CRPD-Sed	CRPD-Sed	CRPD-Ex	CRPD-Ex	
Variable	Baseline	Post 4 weeks	Baseline	Post 4 weeks
Energy (kcal)	2189 ± 689	1306 ± 539*	2466 ± 602	1590 ± 587*
Protein (g)	82 ± 32	71 ± 39	80 ± 36	87 ± 30
Protein (% energy)	15 ± 7	22 ± 6*	13 ± 5	22 ± 7*
Carbohydrate (g)	268 ± 98	52 ± 9*	277 ± 105	51 ± 7*
Carbohydrate (% energy)	49 ± 6	16 ± 5*	45 ± 9	13 ± 4*
Total Fat (g)	202 ± 39	90 ± 35*	117 ± 31	118 ± 27
Total Fat (% energy)	37 ± 9	62 ± 13*	43 ± 11	67 ± 11*
Saturated Fat (g)	37 ± 12	43 ± 17*	34 ± 11	40 ± 14*
Monounsaturated Fat (g)	22 ± 7	38 ± 12*	24 ± 9	42 ± 17*
Polyunsaturated Fat (g)	12 ± 4	14 ± 6	12 ± 6	17 ± 9*
Alcohol (% energy)	2 ± 2	1 ± 2	1 ± 1	1 ± 1
Cholesterol (mg)	443 ± 189	654 ± 289*	398 ± 162	687 ± 279*

Diet and exercise increase BDNF protein content measured in serum. Basal BDNF protein content was significantly increased following CRPD-Sed (+20%; P < 0.05) and CRPD-Ex (+38%; P < 0.05) when compared to baseline. The addition of HIIT exercise to the diet resulted in a significant increase in circulating serum BDNF when compared to CRPD-Sed (Table 2).

**Table 2:** Changes in BDNF protein and cognitive function throughout the study. Values are mean ± SD *Significance between baseline and week 4 of intervention at P ≤ 0.05 †Significance between CRPD-Sed and CRPD-Ex at P ≤ 0.05 Brain-derived neurotrophic factor (BDNF) measured via ELISA in ng/ml; “Cognitive Symptoms & Function” measured via MOS-CFS instrument; “Cognitive Speed & Flexibility” and “cognitive flexibility” measured via Stroop testing. CRPD: Carbohydrate-Restricted Paleolithic-Based Diet

	CRPD-Sed	CRPD-Sed	CRPD-Ex	CRPD-Ex
Variable	Baseline	Post 4 weeks	Baseline	Post 4 weeks
Serum BDNF (ng/ml)	15.4 ± 3.5	18.5 ± 4.6*	15.2 ± 4.3	21.2 ± 6.4*^†^
Cognitive Symptoms & Function (%)	83 ± 6.7	90 ± 5.4*	80 ± 6.1	93 ± 6.0*^†^
Cognitive Speed & Flexibility	146 ± 16.4	125 ± 18.3*	142 ± 15.9	122 ± 12.2*^†^
Cognitive Flexibility	6.4 ± 1.9	3.9 ± 0.8*	5.9 ± 1.2	3.2 ± 0.6*^†^

The Stroop test was used to assess cognitive speed and flexibility. A lower number indicates a faster performance time. The performance outcomes showed significant increases in cognitive speed and cognitive flexibility following the CRPD-Sed (-6%; P < 0.05) and CRPD-Ex (-11%; P < 0.05), when compared to baseline (Table 2). HIIT exercise significantly increased cognitive speed and flexibility beyond diet with sedentary activity.

The MOS-CFS survey was used to assess cognitive symptoms and function. A higher percentage would indicate a stronger self-perceived cognitive functioning. The self-reported outcomes showed a significant increase in cognitive symptoms and function following CRPD-Sed (+8%; P < 0.05) and CRPD-Ex (+16%; P < 0.05), when compared to baseline (Table 2). The addition of HIIT exercise resulted in a significant increase in self-perceived cognitive symptoms and function when compared to CRPD-Sed. No significant differences were found between baselines (week 0 and post-washout), which marked the start of each of the intervention periods.

**Figure 2:**
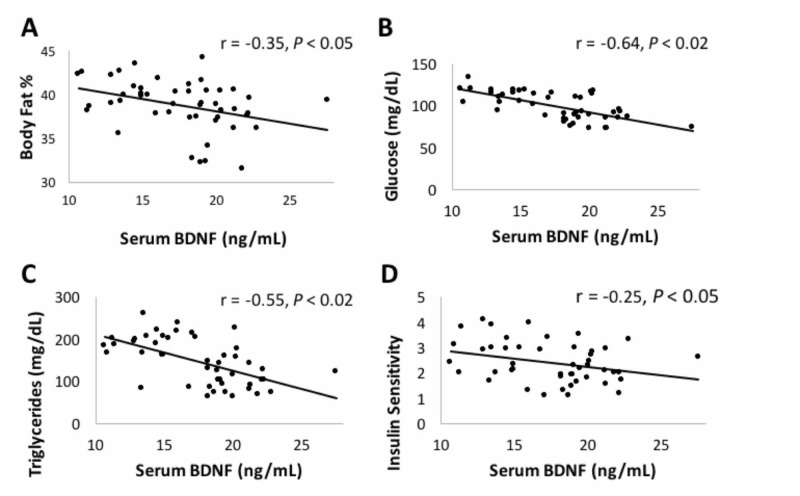
Correlation between circulating BDNF serum levels and cardio-metabolic health markers. An inverse relationship exists between BDNF protein content (ng/mg) and body fat % (Panel A), fasting glucose (Panel B), triglycerides (Panel C), and HOMA-IR (Insulin Sensitivity; Panel D). Each point represents each subject (N = 12) at weeks 0, 4, 8 and 12. BDNF: Brain-derived neurotrophic factor

To better understand the relationship between measured variables, correlations were assessed between BDNF and cardio-metabolic profile markers described elsewhere (paper submitted: Gyorkos, 2019). In Figure 2, each point represents each of the 12 subjects at one of the four data collections times (CRPD-Sed baseline, CRPD-Sed post, CRPD-Ex baseline, CRPD-Ex post), giving 48 data points (n = 48). BDNF inversely correlated with body fat % (r = -0.35; P < 0.05; Figure 2A), fasting glucose (r = -0.64, P < 0.02; Figure 2B), triglycerides (r = -0.55; P < 0.02; Figure 2C), and insulin sensitivity (r = -0.25; P < 0.05; Figure 2D) levels following CRPD-Sed and CRPD-Ex. No correlations were found between serum BDNF and fasting insulin, total cholesterol, high density lipoprotein cholesterol (HDL-C), and low-density lipoprotein cholesterol (HDL-C).

## Discussion

The present study provides novel evidence of a diet and exercise intervention that is capable of increasing BDNF levels and cognitive performance with associated improvements in risk factors for MetS. Further, cognitive benefits were observed when combining high intensity exercise with the diet intervention.

This study revealed that a carbohydrate-restricted diet composed of Paleolithic foods increases circulating BDNF levels and cognition in as little as four weeks in MetS. A strong correlation between BDNF levels and adiposity, insulin sensitivity, and glucose regulation was also observed. Improvements in glucose regulation, insulin sensitivity, inflammation, and body composition have been highly reliable following other restricted diet strategies including dietary-restricted (DR) and carbohydrate-restricted (CR) studies. This current study is consistent with the effects of DR strategy (caloric restriction or intermittent fasting) studies that have resulted in increases in BDNF expression, reductions in glucose and insulin levels, and normalization in weight in human and non-human trials [12].

Findings from this study are also consistent with CR diets that share a common goal of restricting total carbohydrate intake with emphasis on the replacement of energy with fat. CR diets vary as low as 4% to up to 40% of total energy intake as CHO, with the goal of rising ketone body β-hydroxybutyrate to a nutritional ketosis level (typical range of 0.5-3.0 mmol/L). At this low ketosis level, a metabolic shift away from carbohydrates is observed and ketones become the preferred fuel source [20]. These ranges fall in line with the current study, which resulted in ~16% CHO intake and detection of low level nutritional ketosis at 0.5 mmol/L. CR diets have been associated with improvements in lipoproteins, inflammation and metabolic syndrome risk factors [21], as well as neural plasticity, and cognitive function through the actions of neurotrophic factor expression [12].

An alternative to CRPD and other restricted diets is one that is commonly consumed in most industrialized western societies consisting of excess consumption of carbohydrate and fats (high carbohydrate and fat; HCF). HCF diet has been well-established as a means of increasing the risk of developing MetS and associated risk factors. The hallmarks of MetS involve central obesity, insulin resistance and poor glucose control, which have all been found to have deleterious effects on hippocampal volume and learning and memory. Insulin resistance has been marked as one of the key players in the relationship between HCF diet and cognition disruption and is strongly linked to learning and memory [22]. Insulin resistance and other risk factors of MetS are negatively associated with BDNF, the critical mediator of neuronal vitality and function that regulates hippocampal neurogenesis and cognitive performance [23]. HCF diets reduce neuronal plasticity and functional capabilities via reductions in hippocampus BDNF levels [11]. Contrary to HCF, we show here that CRPD is capable of increasing BDNF levels and those levels were correlated with favorable changes in adiposity, insulin sensitivity and glucose regulation.

We further explored the combination of CRPD with HIIT exercise and found that together, diet and exercise were able to increase BDNF levels and cognitive performance significantly when compared to CRPD-Sed. Diet and exercise are both powerful factors that influence energy metabolism and synaptic and cognitive plasticity in the brain, increasing metabotrophic expression, and acting to influence the epigenome to enhance cognitive abilities [24]. Exercise alone is a potent stimulus and capable of reversing the harmful effects of HCF diet on synaptic and neuronal plasticity, presumably acting through the exercise-induced metabotrophic factor, BDNF [25].

BDNF is considered the most susceptible to regulation by exercise of any of the neurotrophic factors [26]. Physical exercise has been consistently shown to increase levels of BDNF mRNA and protein expression in the hypothalamus, striatum and other cortical areas [27]. Further, the increases in serum BDNF levels following exercise have been shown to be intensity-dependent. Ferris et al. showed that exercising at an intensity of 10% above, compared to 20% below ventilatory threshold yielded a larger increase in BDNF levels and cognitive function [28]. Further, shorter bouts of high intensity interval exercise have been shown to elevate BDNF levels above those following intense continuous exercise in healthy subjects [29]. The increased skeletal muscle contractions during HIIT exercise may help to explain the intensity-dependent BDNF elevation observed in this study. It has been proposed that BDNF levels rise in response to skeletal muscle contractions that stimulate the secretion of several muscle gene-products, including FNDC5, a positive regulator of BDNF level in the brain [30].

## Conclusions

In conclusion, carbohydrate-restricted diet and exercise have overlapping benefits on metabotrophic factor expression, metabolic processes, and cognition that are amplified with combining the two different lifestyle factors in those individuals at risk for developing metabolic, cardiovascular and cognitive disease. Future studies are needed to further explore diet-inducing mechanisms of neurotrophic factors, the synergistic mechanisms of lifestyle changes on cognition and metabolic functioning, and critical features of diet and exercise prescription for various populations and conditions.

## References

[1] Huang EJ, Reichardt LF (2001). Neurotrophins: roles in neuronal development and function. Annu Rev Neurosci.

[2] Yan Q, Radeke MJ, Matheson CR, Talvenheimo J, Welcher AA, Felnstein SC (1997). Immunocytochemical localization of TrkB in the central nervous system of the adult rat. J Comp Neurol.

[3] Pedersen BK, Pedersen M, Krabbe KS, Bruunsgaard H, Matthews VB, Febbraio MA (2009). Role of exercise-induced brain-derived neurotrophic factor production in the regulation of energy homeostasis in mammals. Exp Physiol.

[4] Chaldakov G (2011). The metabotrophic NGF and BDNF: an emerging concept. Arch Ital Biol.

[5] Rios M, Fan G, Fekete C (2001). Conditional deletion of brain-derived neurotrophic factor in the postnatal brain leads to obesity and hyperactivity. Mol Endocrinol.

[6] Takei N, Furukawa K, Hanyu O, Sone H, Nawa H (2014). A possible link between BDNF and mTOR in control of food intake. Front Psychol.

[7] Han JC, Liu Q-R, Jones M (2008). Brain-derived neurotrophic factor and obesity in the WAGR syndrome. N Engl J Med.

[8] Chaldakov GN, Fiore M, Stankulov IS (2004). Neurotrophin presence in human coronary atherosclerosis and metabolic syndrome: a role for NGF and BDNF in cardiovascular disease?. Prog Brain Res.

[9] Krabbe KS, Nielsen AR, Krogh-Madsen R (2007). Brain-derived neurotrophic factor (BDNF) and type 2 diabetes. Diabetologia.

[10] Nakagawa T, Tsuchida A, Itakura Y (2000). Brain-derived neurotrophic factor regulates glucose metabolism by modulating energy balance in diabetic mice. Diabetes.

[11] Molteni R, Barnard R, Ying Z, Roberts C, Gómez-Pinilla F (2002). A high-fat, refined sugar diet reduces hippocampal brain-derived neurotrophic factor, neuronal plasticity, and learning. Neuroscience.

[12] Araya AV, Orellana X, Espinoza J (2008). Evaluation of the effect of caloric restriction on serum BDNF in overweight and obese subjects: preliminary evidences. Endocrine.

[13] Cho H, Kim J, Kim S, Son YH, Lee N, Jung SH (2012). The concentrations of serum, plasma and platelet BDNF are all increased by treadmill VO2max performance in healthy college men. Neurosci Lett.

[14] Saucedo Marquez CM, Vanaudenaerde B, Troosters T, Wenderoth N (2015). High-intensity interval training evokes larger serum BDNF levels compared with intense continuous exercise. J Appl Physiol.

[15] Grundy SM, Brewer HB Jr, Cleeman JI, Smith SC Jr, Lenfant C (2004). Definition of metabolic syndrome: report of the National Heart, Lung, and Blood Institute/American Heart Association conference on scientific issues related to definition. Circulation.

[16] Johnson R, Driscoll P, Goran M (1996). Comparison of multiple-pass 24-hour recall estimates of energy intake with total energy expenditure determined by the doubly labeled water method in young children. J Am Diet Assoc.

[17] Karege F, Schwald M, Cisse M (2002). Postnatal developmental profile of brain-derived neurotrophic factor in rat brain and platelets. Neurosci Lett.

[18] Revicki DA, Chan K, Gevirtz F (1998). Discriminant validity of the Medical Outcomes Study cognitive function scale in HIV disease patients. Qual Life Res.

[19] Volek JS, Sharman MJ, Gómez AL, DiPasquale C, Roti M, Pumerantz A, Kraemer WJ (2004). Comparison of a very low-carbohydrate and low-fat diet on fasting lipids, LDL subclasses, insulin resistance, and postprandial lipemic responses in overweight women. J Am Coll Nutr.

[20] Maalouf M, Rho JM, Mattson MP (2009). The neuroprotective properties of calorie restriction, the ketogenic diet, and ketone bodies. Brain Res Rev.

[21] Volek JS, Phinney SD, Forsythe CE (2009). Carbohydrate restriction has a more favorable impact on the metabolic syndrome than a low fat diet. Lipids.

[22] McNay EC, Ong CT, McCrimmon RJ, Cresswell J, Bogan JS, Sherwin RS (2010). Hippocampal memory processes are modulated by insulin and high-fat-induced insulin resistance. Neurobiol Learn Mem.

[23] Castrén E, Berninger B, Leingärtner A, Lindholm D (1998). Regulation of brain-derived neurotrophic factor mRNA levels in hippocampus by neuronal activity. Prog Brain Res.

[24] Vaynman S, Ying Z, Wu A, Gomez-Pinilla F (2006). Coupling energy metabolism with a mechanism to support brain-derived neurotrophic factor-mediated synaptic plasticity. Neuroscience.

[25] Molteni R, Wu A, Vaynman S, Ying Z, Barnard RJ, Gómez-Pinilla F (2004). Exercise reverses the harmful effects of consumption of a high-fat diet on synaptic and behavioral plasticity associated to the action of brain-derived neurotrophic factor. Neuroscience.

[26] Gomez-Pinilla F (2011). The combined effects of exercise and foods in preventing neurological and cognitive disorders. Prev Med.

[27] Knaepen K, Goekint M, Heyman EM, Meeusen R (2010). Neuroplasticity - Exercise-induced response of peripheral brain-derived neurotrophic factor. Sport Med.

[28] Ferris LT, Williams JS, Shen C-L (2007). The effect of acute exercise on serum brain-derived neurotrophic factor levels and cognitive function. Med Sci Sports Exerc.

[29] Saucedo Marquez CM, Vanaudenaerde B, Troosters T, Wenderoth N (2015). High-intensity interval training evokes larger serum BDNF levels compared with intense continuous exercise. J Appl Physiol.

[30] Wrann CD, White JP, Salogiannnis J (2013). Exercise Induces Hippocampal BDNF through a PGC-1α/FNDC5 Pathway. Cell Metab.

